# Rome consensus conference - statement; human papilloma virus diseases in males

**DOI:** 10.1186/1471-2458-13-117

**Published:** 2013-02-07

**Authors:** Andrea Lenzi, Vincenzo Mirone, Vincenzo Gentile, Riccardo Bartoletti, Vincenzo Ficarra, Carlo Foresta, Luciano Mariani, Sandra Mazzoli, Saverio G Parisi, Antonio Perino, Mauro Picardo, Carla Maria Zotti

**Affiliations:** 1 Institute/Department of Endocrinology, La Sapienza University, Rome, Italy; 2 Institute/Department of Urology, Federico II University, Naples, Italy; 3 Institute/Department of Urology, La Sapienza University, Rome, Italy; 4 Department of Urology, Santa Maria Annunziata Hospital, Florence, Italy; 5 Department of Urology, University of Padua, Padua, Italy; 6 Department of Histology, Microbiology and Medical Biotechnologies, Centre for Male Gamete Cryopreservation, University of Padua, Padua, Italy; 7 Department of Obstetrics and Gynaecology, Regina Elena National Cancer Institute, Rome, Italy; 8 Centre Responsible, Sexually Transmitted Disease Centre, Santa Maria Annunziata Hospital, Florence, Italy; 9 Department of Histology, Microbiology and Medical Biotechnologies, University of Padua, Padua, Italy; 10 Head of Obstetrics and Gynecology Department, University of Palermo, Palermo, Italy; 11 Laboratory of Skin Physiopathology San Gallicano Dermatological Institute IRCCS, Rome, Italy; 12 Department of Public Health and Microbiology, University of Turin, Turin, Italy

**Keywords:** HPV-related diseases, Vaccination, Males, Consensus conference, Policy

## Abstract

**Background:**

Human Papillomavirus (HPV) is a very resistant, ubiquitous virus that can survive in the environment without a host. The decision to analyse HPV-related diseases in males was due to the broad dissemination of the virus, and, above all, by the need to stress the importance of primary and secondary prevention measures (currently available for women exclusively). The objective of the Consensus Conference was to make evidence-based recommendations that were designed to facilitate the adoption of a standard approach in clinical practice in Italy.

**Methods:**

The Sponsoring Panel put a series of questions to the members of the Scientific Committee who prepared a summary of the currently available information, relevant for each question, after the review and grading of the existing scientific literature. The summaries were presented to a Jury, also called multidisciplinary Consensus Panel, who drafted a series of recommendations.

**Results:**

The prevalence of HPV in males ranges between 1.3–72.9%;. The prevalence curve in males is much higher than that in females and does not tend to decline with age. Women appear to have a higher probability of acquiring HPV genotypes associated with a high oncogenic risk, whereas in males the probability of acquiring low- or high-risk genotypes is similar. The HPV-related diseases that affect males are anogenital warts and cancers of the penis, anus and oropharynx. The quadrivalent vaccine against HPV has proved to be effective in preventing external genital lesions in males aged 16–26 years in 90.4%; (95%; CI: 69.2–98.1) of cases. It has also proved to be effective in preventing precancerous anal lesions in 77.5%; (95%; CI: 39.6–93.3) of cases in a per-protocol analysis and in 91.7%; (95%; CI: 44.6–99.8) of cases in a post-hoc analysis. Early ecological studies demonstrate reduction of genital warts in vaccinated females and some herd immunity in males when vaccine coverage is high, although males who have sex with males gained no benefit at all. Males with an immunodeficiency disease are at greater risk of developing disease. Infertility seems to be caused by HPV in some cases. Studies demonstrate vaccination to both genders can be more efficacious and social equity matters are to be taken into consideration.

**Conclusions:**

The Jury made Recommendations based on the scientific evidence presented by the Scientific Committee. Accordingly, for prevention purposes and social fairness and equality, as both sexes are affected by the disease, the vaccination of 12-year-old males against HPV should be recommended in order to guaranty protection to everyone. Aspects related to healthcare policy and economic sustainability, are to be discussed by respective public system representatives. More campaigns to raise awareness through all institutional channels are needed, not only regarding anogenital warts, but for HPV-related diseases in general in males in accordance to new scientific evidences.

## Introduction

The Human Papillomavirus (HPV) is a very resistant, ubiquitous virus that can survive in the environment without a host [1]. It may remain inactive for a long time and produce asymptomatic infections of the skin. It can be transmitted from one individual to another directly (by sexual contact) or indirectly.

The dynamics of heterosexual transmission of HPV (the major route of infection) are still being investigated and their relative importance has not been established yet. The ability of HPV to survive for a long time on various surfaces, objects, and parts of the body that have had contact with infected mucous membranes makes potential transmission to the genitals via the hands, the perigenital skin (the scrotum or pubis) or even inanimate objects possible [2].

HPV has been found on the skin and the mucous membranes of individual exposed to the virus, and causes both anogenital and oropharyngeal infections. In women, the prevalence of the different genotypes of HPV has been determined and analyzed within the setting of cytologic examinations with the purpose of screening for and diagnosing precancerous and cancerous lesions. In men, the first studies focused on HPV infection and the risk of cancer in the homosexual population. More recently, studies on the importance and clinical consequences of HPV infection have been extended to the heterosexual male population, and have also examined the role of males in the transmission of HPV to women.

The frequency of HPV infection in various groups of male subjects was analyzed in a systematic review of the literature, which included 40 papers published from 1999 to 2006 [3]. Overall, the prevalence of HPV in men ranged from 1.3%; to 72.9%;, and it exceeded 20%; in 56%; of the studies analyzed. In another review, carried out by Partridge et al. on studies conducted from 1991 to 2005, the prevalence of HPV in heterosexual males ranged from 3.5%; to 45%; [4].

One finding is difficult to explain: the prevalence of HPV infection in men does not vary among different age groups, although its incidence is similar in both genders and its clearance is fairly rapid. A possible explanation is that repeated infections occur during sexual activity and persist for variable periods of time. However, a longitudinal assessment of a cohort of males who are followed up in the long term is needed to provide more information on this phenomenon. The following points have become apparent with respect to HPV-related diseases in males:

– - Several issues of the diseases are controversial and should be addressed by adopting a multidisciplinary and multiprofessional approach;

– - Such diseases are a public health matter and have aroused the interest of healthcare professionals and users (patients/citizens), with potential impacts on clinical practice;

– - The scientific data on these diseases are incomplete, but nevertheless enable the drafting of a reliable consensus document that is based on expert opinions;

– - Exhaustive information on HPV-related diseases in males is not available, so it is important to make recommendations for further research;

– - A document that lays down recommendations for the prevention and management of HPV-related diseases in males, may reduce variability in healthcare and, possibly, related costs.

The decision to analyze HPV-related diseases in males by establishing a Consensus Conference (CC) was due to the broad dissemination of the virus, and, above all, by the need to stress the importance of measures for primary prevention and organized screening programs, which, at present, are the only tools we have to reduce the incidence of HPV-related diseases. It is worth noting that in clinical practice there are neither validated tests nor screening programs for HPV in males at the moment.

The objective of the CC was to make evidence-based recommendations, starting from a review of existing literature that was designed to assess the best scientific evidence available currently.

## Methods

A CC is one of the tools that enable agreement to be reached, through a formal process, on healthcare issues that are particularly complex and controversial. The outcome facilitates a uniform approach to such issues in clinical practice.

### Consensus Conference protocol

The consensus conference on Human Papillomavirus Diseases in Males was called by a Sponsor Panel (SP), which included representatives of three Italian scientific societies: SIA – (Italian Society of Andrology), SIAMS (Italian Society of Andrology and Sexual Medicine), SIU (Italian Society of Urology). They appointed ten acknowledged experts in the field to set up the Scientific Committee (SC) of experts.

In January 2010 the SP gathered the Scientific Committee to submit four specific predefined questions of importance to better understand HPV in males about which the SC was asked to draw a synthesis of the available knowledge starting from a revision of the scientific literature on the topics.

The expert panel revised the scientific literature, classifying it into the following areas:

– - HPV epidemiology

– - Natural history of HPV infection

– - HPV burden of disease

– - HPV prevention strategies

– - HPV vaccines

– - Social impact of HPV diseases

Inclusion criteria and paper selection process were established in accordance with the Sponsor Panel and the Panel of experts. To be included in the review, each paper had to: a) contain data from males or separately presented from females; and b) contain country-specific/racial/sexual-habits data for male subjects or c) report on HPV prevalent in males or d) report on incidence of HPV–related events in males or f) report on relationship between HPV and fertility or g) report results on diagnostic testing methods on males or separately presented from females.

Studies that did not meet criteria a) and b) and at least one of criteria c), d), f), g) were ineligible. Literature collection started in January 2010; four coordinators (one for each question of the Consensus Conference) were encharged of retrieving all the relevant documents through November (2010), following the up-reported selection criteria. Longitudinal, retrospective and cross-sectional studies, as well as randomized clinical trials were considered, upon availability. The search process used the following database to identify articles published until 2010 included:

Medline, Embase, Cochrane Controlled Trials Register, PubMed (National Library of Medicine), and the Cochrane Library were searched to identify articles published and the following keywords: HPV or Papillomavirus” OR “HPV Vaccination” OR “HPV infection/epidemiology” OR “Papillomavirus natural history” And (Italy and (prevalence or incidence) OR (efficacy, burden, social equity, fertility, prevention) And Male.

**Figure 1:**
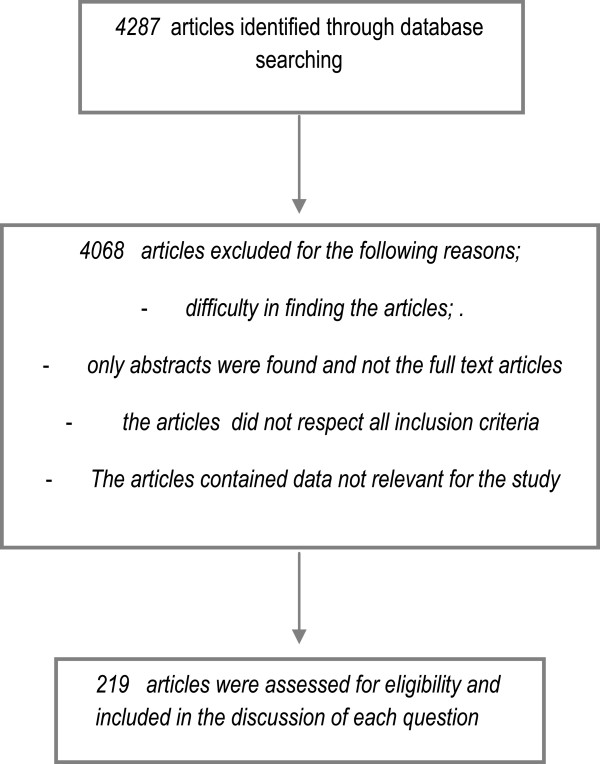
Flow of information to describe the results of the entire process.

See flow diagram in Figure 1 that illustrates the results of the entire process.

All the eligible papers were used by the four coordinators to argue on their specific question and were included in the references section. Among the eligible papers, those supporting the question conclusions were reviewed by an independent methodological expert and assessed with the quality rating reports in Table 1.

**Table 1:** Grading of scientific evidence (5)

**Grading**	**Definitions**
1++	High quality meta-analyses, systematic reviews of RCTs, or RCTs with a very low risk of bias
1+	Well conducted meta-analyses, systematic reviews of RCTs, or RCTs with a low risk of bias
1–	Meta-analyses, systematic reviews or RCTs, or RCTs with a high risk of bias
2++	High quality systematic reviews of case–control or cohort studies or high quality case–control or cohort studies with a very low risk of confounding, bias, or chance and a high probability that the relationship is causal
2+	Well conducted case–control or cohort studies with a low risk of confounding, bias, or chance and a moderate probability that the relationship is causal
2–	Case–control or cohort studies with a high risk of confounding, bias, or chance and a significant risk that the relationship is not causal
3	Non­analytic studies, eg case reports, case series
4	Expert opinion

*RCT *Randomized and Controlled Trials.

During year 2010 the SP called 3 meetings with the SC in order to examine the various revisions of the document drawn together from scientific evidence for the presentation of the results.

The final document with the results was presented on 22nd November 2010, by the SP and the SC to a Jury called also a multidisciplinary Consensus panel of experts composed of the following: 1 representative of the ethic committee, 1 oncological gynecology, 1 pediatrician, 2 specialist of public health and epidemiology, 1 representative of the Italian Medicines Agency (AIFA), 1 representative of National Institute of Health (ISS), 1 representative of the Ministry of Equal Opportunities, 1 Member of “Cittadinanzattiva” (Active citizenship – an Italian non-profit organization of citizens).

Each question posed to the SC was discussed during the day of the consensus.

On the basis of the document which was presented and the lectures held by the SC during the consensus meeting, the Jury reached a general agreement about the information provided and how to answer to the key questions, drawing from each questions recommendations based on their observations.

The document with the results of the work and there commendations of the Jury was printed after the Consensus Conference.

### Grading of scientific evidence

Various methods have been designed to assess the robustness of clinical recommendations. At present, an international method is being developed, although it will be difficult to reconcile different approaches and techniques and include them all in a single assessment system In the present case, the outline published in the British Medical Journal in 2001 was followed (Table 1) [5].

### Questions put by the SC

To carry out an in-depth review and reach an agreement on the prevention of HPV-related diseases in males, which is a complex healthcare issue that involves a number of grey areas, the following list of questions was drawn up:

Question no. 1: What is the impact of HPV-related diseases in males?

Question no. 2: What role does a positive test for HPV infection in a partner play in the problems associated with HPV-related diseases?

Question no. 3: What method of prevention of HPV-related diseases could significantly reduce their impact on the male population?

Question no. 4: Could primary prevention of HPV-related diseases in men reduce HPV-related diseases in their partners?

## Results

### Question no. 1: What is the impact of HPV-related diseases in males?

A) Prevalence of the infection [3] – Grading = 2++

In a systematic review by Dunne et al. on prevalence of HPV in men [3] it was found to be as high as 72.9%; and the median probability of transmission per sex act was around 40%;. Table 2 reports the results of prevalence studies in which the polymerase chain reaction (PCR) was used to detect the presence of the virus in multiple samples collected from different anatomical sites that were deemed to be most suitable for the purpose (e.g. the glans, sulcus and scrotum). The study populations varied, and included the general population (workers, students), patients attending STD clinics, healthy participants, and the partners of women with HPV-related diseases (CIN1+). Studies that didn’t took into consideration partners HPV positivity and which included university students, military recruits, and healthy, sexually active males, prevalence ranged between 6%;–45%;. The mean prevalence of positive tests in the partners of women with cervical intraepithelial neoplasia (CIN) lesions was higher (50–70%;), whereas among males recruited at clinics for sexually transmitted diseases (STDs), the prevalence of positive tests was around 45%;. One of the most recent and significant studies, in terms of population sampled, conducted by Giuliano et al. [6] included 1.160 men in three countries, reporting a total prevalence of 65.2%; HPV positivity. Although not a review, this study alone, adds more subjects than all previous studies placed together, making it one of the most statistical significant studies on the subject.

B) The prevalence of infection by age [6] – Grading = 2+

**Table 2:** Main prevalence studies conducted using the PCR method

**Population type (n° males examined)**	**Country**	**Prevalence of positive HPV DNA (PCR test)**	**Prevalence of positive HPV DNA tests (high-risk genotypes)**
Population (290)	USA	30.0%;	16.6%;
Giuliano et al., 2007 [7]			
Military recruits (285)	Finland	16.5%;	- -
Hippelainen et al., 1993 [8]			
University students (317)	USA	32.8%;	14.5%;
Weaver et al., 2004 [9]			
Students and industrial workers (114)	Mexico	36.0%;	16.7%;
Lazcano-Ponce et al., 2001 [10]			
Military recruits (337)	Denmark	33.8%;	- -
Kjaier et al., 2005 [11]			
Military men (1030)	Mexico	44.6%;	34.8
Lajous et al., 2005 [12]			
University students (381)	South Korea	8.7%;	4.2%;
Shin et al., 2004 [13]			
Subjects attending STD clinics (235)	Sweden	13.2%;	8.1%;
Wikstrom et al., 2000 [14]			
Subjects attending STD clinics (198)	Denmark	44.9%;	- -
Svare et al., 2002 [15]			
Subjects attending STD clinics (393)	US	28.2%;	12.0%;
Baldwin et al., 2003 [16]			
Subjects attending STD clinics (85)	Netherland	28.2%;	- -
Van Doornum et al., 1994 [17]			
Subjects attending STD clinics (204)	Japan	5.9%;	5.9%;
Takahashi et al., 2005 [18]			
Males with female partner affected by CIN (119)	Netherland	59.0%;	55.4%;
Bleeker et al., 2002 [19]			
Husbands of women recruited in case–control studies on carcinoma of the cervix (1143)	Thailand, Philippines, Brazil, Columbia, Spain	16.0%;	- -
Franceschi et al., 2002 [20]			
Males with female partner affected by CIN (181)	Netherland	72.9%;	58.6%;
Bleeker et al., 2005 [21]			
Males with female partner affected by CIN (77)	Finland	9.1%;	- -
Hippelainen et al., 1994 [22]			
Males with female partner affected by HPV (50)	Brazil	70.0%;	- -
Nicolau et al., 2005 [23]			
Males through 18–70 years from one of the three sampling sites (1,160)	Brazil	65.2%;	12.0%; oncogenic types only
Giuliano et al., 2008 [6]	Mexico		
	USA		

*PCR *Polymerase Chain Reaction, *CIN *Cervical Intraepithelial Neoplasia.

In males, the prevalence of HPV does not differ significantly among age group as it does in females [6]. Indeed, prevalence in males remains high (50–70%;) throughout their lifetime, without any substantial decline with age. In contrast, in women, the prevalence curve is consistently bimodal (with some geographical variability): there is a peak after first sexual intercourse up to the age of 25–30 years, followed by a decline up to the perimenopausal age, when a second peak is often observed [24]. This pattern has been observed in Italy too, according to an epidemiological study conducted in Turin [25]. In the Turin study, the first peak of HPV prevalence corresponded to 13–14%; (with a manifestation occurring up to the age of 39 years), after which the prevalence decreased to 5%; at the age of 45 years and then increased to 8%; during the perimenopausal period. Another important difference between males and females is the probability of acquiring an HPV genotype that is associated with a high risk (HR) or a low risk (LR) of oncogenicity (HR-HPV and LR-HPV, respectively). Women seem to have a higher probability of acquiring an HR-HPV [26], whereas the probability of acquiring the two types of HPV appears to be similar in males (the 24-month cumulative incidence was 47.9%; for HR vs 46.6%; for LR) [27].

C) Incidence and clearance of the infection [27] – Grading = 2+

The natural course of the disease has not been investigated properly in males from an epidemiological point of view, namely, by establishing rates of acquisition and time to clearance of HPV infection. A few prospective studies have estimated the probability of acquiring the infection for heterosexual males. In a population of 290 male participants aged between 18 and 44 years (of whom 88%; claimed to be heterosexual), who were followed up for a mean period of 15.5 months [27], the overall prevalence of HPV infection (any genotype) was 30%;, the period prevalence (positive test at any time in the study) was 52.8%;, and the incidence of infection amounted to 29.4 per 1,000 person–months. The incidence of new infections, as measured in males with a negative HPV test at recruitment, was 42.3 per 1,000 person–months. Using Kaplan–Meier survival curves, it was estimated that the rate of acquisition of new infections was 29.2%; over 12 months (19%; for HR-HPV vs 16%; for LR-HPV), and the rate of acquisition of the HR genotypes 16 and 18 (5.1%; of new infections) was higher than that of LR genotypes 6 and 11 (3.4%; of new infections). The mean clearance time (defined as the time to elimination of 50%; of all infections) was estimated to be 5.9 months (95%; CI: 5.7–6.1). HPV DNA was no longer detectable in 75%; of infected subjects 12 months from onset of the infection, without any differences between HR-HPV and LR-HPV, after 5.9 months [27].

The study by Partridge [27], which was conducted in a population of 240 male university students aged 18–23 years, yielded a prevalence of 25.8%; and estimated a cumulative incidence at 24 months of 62.4%; (95%; CI: 52.6–72.2) for all HPV genotypes. The incidence of HR-HPV and LR-HPV genotypes was similar (approximately 48%;). The glans, the shaft of the penis, and the scrotum had the same probability of being infected (44%;); whereas the positive test rate of urinary samples was much lower (7–8%;) [27].

D) HPV-related diseases in males

The HPV-related diseases that occur in males are genital warts and cancers of the penis, anus, oral cavity, and oropharynx (carcinomas of the head and neck).

➣ Anogenital warts [7,28,29] (grading = 2–, 3, 2+, respectively)

In most cases (90%;), anogenital warts are caused by HPV genotypes 6 and 11 [7]. In Italy, the HPV infection and genital warts is not subject to mandatory reporting, according to an investigation at 12 STD centres attended by a high-risk population, genital warts were diagnosed in 33%; of subjects, 73%; of whom were male. The mean age and standard deviation (SD) of the male population was 33.6 (±11.0) years, whereas the mean age (SD) of the female population was 30.9 (±10.9). The distribution by age bracket showed that the proportion of anogenital warts was higher in the 14–25-year age group than in the older age brackets, in both males and females. In Italy, genital warts in STD clinics showed that in males, 89.9%; of anogenital warts were diagnosed in self declared heterosexual men and 10.1%; in self declared bisexuals or homosexuals [28]. Data showed that in Italy through STD clinics, genital HPV infections is the most diagnosed STI in Italy, this study is important as it is the only study on genital warts incidence as it is not a reportable disease. Genital warts show a high spread among young people both in males and females.

Having ≥ 2 sexual partners in the last 6 months (reported by 50.3%; of males) and the presence of other concomitant STDs (recorded in 17.1%; of males) were risk factors [28]. In a study conducted in Italy in 2008, it was estimated that the number of cases of anogenital warts per year in women aged between 14 and 64 years was approximately 120,000 (with a prevalence that corresponded to 0.6%;) [28,29].

➣ Cancer of the penis [30-32] (grading = 2–, 2–, 2–, respectively)

Cancer of the penis is a rare tumor in Western countries. It has been estimated that around 1,000 cases of HPV-related cancer of the penis occur in Europe every year. HPV DNA is detected in approximately 40–50%; of all cases of cancer of the penis, and seroepidemiological studies have shown that the main genotypes involved are HPV 16 and 18 [30-32]. No studies are available in Italy.

➣ Cancer of the anus [33,34] (grading = 2–, 2–, respectively)

The HPV virus is detected in 80%; of cancerous lesions of the anus, and genotype 16 is the subtype that is involved most commonly (87%;). In the USA, the incidence of cancer of the anus increased considerably between 1970 and 2000. Its incidence is particularly high in homosexual males and the risk is increased even further in the presence of human immunodeficiency virus (HIV) infection. It has been estimated that, in Europe, something like 2,000 cases of cancer of the anus occur in males every year [33,34].

➣ Carcinomas of the head and neck [16] (grading = 2–)

The overall prevalence of HPV DNA in carcinomas of the head and neck is approximately 26%; and it reaches a peak of 36%; in cancer of the oropharynx. HPV-16 is the genotype involved most commonly (60–80%;). Furthermore, a significant association has been found between HPV in the oral cavity and sexual habits [16].

E) HPV and fertility [35-38] – Grading = 3, 3, 2–, and 3, respectively

Preliminary studies show not only that HPV is present in semen, but that it might reduce sperm motility probably causing reduced fertility in males. Moreover, the virus might interfere with the development of the embryo in animal models according to some studies [35-38]. HPV might be a cause of infertility, even so, further studies are needed in order to clarify its relationship with fertility.

F) Diagnosis of HPV in males [39-41] – Grading = 2+, 2++, 2–, respectively

At the moment, there is no generally accepted and validated test for screening HPV in males in the clinical practice. However, there is a general consensus on when diagnostic testing should be performed, which may be summarized as follows [39-41]:

1. When the patient has a partner who is HPV positive or has an HPV-related disease;

2. When HPV-related clinical manifestations are present;

3. When the patient has sex with men.

At present, molecular methods are used most commonly, both to detect infection and to identify the virus genotype. The tests are based on amplification methods (PCR) with hybridization [42]. Cell samples for the HPV test may be collected from multiple sites (penis shaft; balano-preputial sulcus; glans; navicular fossa of the urethra; scrotum; the pubic, perianal, and anal areas; the oral cavity; the oropharynx; and the larynx), possibly from suspicious lesions [31,43].

For males, a test that identifies not only HR-HPV but also LR-HPV and genotypes associated with intermediate risk or that have not been classified yet would be useful, because it may enable differential diagnoses to be made between benign and malignant lesions and those related or not related to HPV (e.g. molluscum contagiosum) [44-46].

G) Follow-up of male patients with HPV-related diseases [47,48] – Grading = 4, and 1 (according to the Guidelines of the Italian Society of Virology)

The following investigations are suggested for the follow-up of HPV-related diseases:

➣ According to the site involved, benign lesions should be checked clinically with genitoscopy and/or anoscopy every 4 months for the first year, as appropriate. Molecular analysis to screen for HPV should be carried out with the same frequency;

➣ In subjects at risk (those with neoplastic lesions or with a history of HPV-related neoplasias), testing for HPV DNA should be carried out every 4 to 6 months;

➣ In subjects with a greater risk of infection and disease development (immunodepressed, HIV-positive and/or homosexual subjects), testing for HPV should be carried out every 8 to 12 months;

➣ In asymptomatic HPV-positive subjects, laboratory follow-up is recommended after 8 to 12 months to assess the persistence or clearance of the infection and to check whether any lesions have developed.

#### Question conclusions

According to the answers given by the SC, Impact of HPV-related diseases seem to be high in males. Not the same burden as in women for high risk HPV types related lesions but more when taking into account only low grade HPV related lesions such as genital warts. Prevalence seems to be higher than in women irrespective of age and no standardized HPV test guidelines are recommended as in women. Preliminary fertility studies in men seem to show that HPV might have an important role, more studies and data need to be done in order to prove HPV relation with fertility.

#### Conclusive considerations of the consensus panel

➣ More prevalence studies in males should be conducted. In Italy, notification of HPV related diseases such as genital warts are not mandatory, more epidemiological data is needed in order to evaluate disease impact.

➣ More scientific studies should be made about data on the standardized collection of biological samples and method of testing used for HPV detection in males in order to implement in the future standard recognized sampling and diagnostic tests in eventual prevention and screening programs.

➣ Further research on the issue of HPV and fertility worldwide is required in order to fully understand if it is related and affected by HPV and in what measures HPV vaccination might reduce this.

### Question no. 2: What role does a positive test for HPV infection in a partner play in the problems associated with HPV-related diseases?

A) Transmission potential of the virus [1,2,27,49] – Grading = 2++, 2++, 2++, and 2+, respectively

HPV is a highly contagious virus that is widespread in the environment. It is transmitted mainly through sexual contact. However, indirect secondary modes of transmission also exist (contact of skin with skin, hetero-inoculation mediated by the hands, contact with underwear or inanimate objects) [1,2,27,49]. To assess the role that males might have in the transmission of HPV to female partners, studies on couples appear to be more relevant than population studies. Interestingly enough, most studies are focused on long term relationship, or relationships on which the female partner is affected of an HPV lesion such as CIN, little is known about recently established relations at an early age, that can help understand HPV dynamics, natural history and transmission models in young couples. HPV concordance data is variable throughout different studies. In Studies conducted in couples that had a mean duration time of 10 years of relationship, concordance of at least one HPV type in couples that were both positive for HPV was of 57.8%; [21] which is more prevalent than expected by chance, other studies conducted in Italian couples, demonstrate also high HPV concordance in infected couples of around 45%; [50]. Both studies show high HPV infection rates irrespective of HPV type, especially when the female partner is already infected, concordance rates might be important to understand transmission dynamics that are important for persistent infections and development of lesions. In recently established couples, according to Burchell et al., the rate of HPV infections was around 64%;, of which concordance was found in 41%; of the heterosexual couples [51], even in a young recently made couple (aged 18–24) HPV infection and concordance was higher than expected by chance. In time new most newly made couples will probably clear infection, even so high prevalence and concordance with time, like in long term relationships, might explain persistent infection dynamics and explain in part increased lesion development risks. Genotype concordance in couples could depend on several variables: behavioural variables (frequency, type, duration of sexual contact and protection measures), biological variables (different susceptibilities and clearance abilities of tissues in the two genders), and probably others that have not yet been identified.

B) Transmission dynamics [50,52-54] – Grading = 2–, 2++, 2++, and 2+, respectively

Transmission of the virus from one partner to another varies also according to sexual practices. Many male partners may be classified as «healthy carriers» who unknowingly serve as an asymptomatic reservoir and could contribute to the development of HPV-related diseases in women [52-54].

C) Risk factors and monitoring [27,55-58] – Grading = 2++, 2–, 2–, 2+, and 4, respectively

In males, age does not appear to be related clearly to the acquisition of the infection. In general, sexual habits (duration of sexual activity and number of recent partners) correlate with the presence of infection, in both males and females. Individual factors (age at first sexual intercourse, number of partners while sexually active, smoking habits, and alcohol consumption) or factors related to the female partner (anal sex, partner with a history of STD) are not related consistently and significantly to the acquisition of infection and its presence in males [3,4,27]. In addition, a state of immunodeficiency (due to HIV infection) is a risk factor for infection of HPV [55-57]. Every male sexual partner of a woman affected by anogenital warts should be examined to detect and, if necessary, treat any esophytic HPV-related lesions. It is important to extend monitoring beyond the mean incubation period (3 months) [58]. At present, testing for HPV (through PCR) in asymptomatic males with a negative examination of the penis (penoscopy) is not recommended, even when the female partner has had a positive cytological examination [58].

#### Question conclusions

Based on the previous statements and data, HPV DNA sero status seem to play a fundamental role in HPV transmission and diseases development between partners. In males, there are several risk factors that play a fundamental role in HPV infection and development of the disease such as immunodeficiency disease.

#### Conclusive considerations of the consensus panel

➣ Awareness campaigns should be implemented not only for anogenital warts but for HPV-related diseases in general, through all institutional channels, in order to increase HPV knowledge and reduce, when possible, HPV transmissibility at a population level.

➣ Subjects with an immunodeficiency status, such as HIV, have a greater risk of HPV infection; prevention strategies should also target high risk populations.

### Question no. 3: What method of prevention of HPV-related diseases could significantly reduce their impact on the male population?

A) Primary Prevention [59-68] – Grading = 1++, 1++, 1++, 1++, 1++, 1++, 1++, 1+, 1++, and 2+, respectively.

Primary prevention through vaccination has proved to be very effective in preventing precancerous lesions and cancers of the cervix, vulva, and vagina, as well as anogenital warts, in women up to the age of 45 years [59-66]. The quadrivalent vaccine is effective against HPV genotypes 6, 11, 16, and 18. In Europe, the vaccine is indicated for the prevention of cervical cancer, precancerous lesions of the cervix, vulva, and vagina, and also for genital warts in women aged between 9 and 45 years [69].

Clinical trials have shown that the quadrivalent vaccine is effective in preventing external genital lesions (anogenital warts, as well as penile and perineal lesions) in males aged between 16 and 26 years in 90.4%; (95%; CI: 69.2–98.1) of cases [67]. On the basis of these results, in October 2009, the US Food and Drug Administration (FDA) approved the extension of the indications for the quadrivalent vaccine to males up to the age of 26 years, for the prevention of anogenital warts. In addition to the USA, another seven countries have approved the extension of the indications for the quadrivalent vaccine to males up to the age of 26 years for the prevention of anogenital warts [70].

At present, efficacy data on the quadrivalent vaccine with respect to the prevention of external genital and anal precancerous lesions are available (Table 3). In a subpopulation of homosexual males aged between 16 and 26 years, population at a higher risk of anal precancerous lesions, the quadrivalent vaccine was effective in 77.5%; (95%; CI: 39.6–93.3) of cases in preventing precancerous anal lesions (per-protocol analysis); furthermore, in a post-hoc analysis, the efficacy rate was 91.7%; (95%; CI: 44.6–99.8) [71,72] specifically from HPV types 16 and 18.

**Table 3:** Efficacy of the quadrivalent vaccine in preventing external genital lesions and AIN (per-protocol population)

	**Giuliano **[67]	**Palefsky **[71]	**Goldstone **[72]
Population	Per-protocol	Per-protocol	Per-protocol*
	(16–26 years)	(16–26 years)	(16–26 years)
External genital lesions	90.4%;		
	(95%; CI: 69.2–98.1)		
AIN		77.5%;	91.7%;
		(95%; CI: 39.6–93.3)	(95%; CI: 44.6–99.8)
Anogenital warts	89.4%;		
	(95%; CI: 65.5–97.9)		

* Post-hoc analysis. *AIN *Anal intraepithelial neoplasia.

Notes of importance after the consensus:

In accordance to the importance and recommendations already stated by the CC document about HPV in males, in late 2010 and 2011, on the basis of vaccine efficacy data against HPV-related anal precancerous lesions, in December 2010, the FDA approved the extension of the indications for the quadrivalent vaccine to the prevention of anal cancer and anal intraepithelial lesions in males and females aged between 9 and 26 years [73]. In addition, the results for efficacy related to the prevention of anal cancer and anogenital warts in males aged 16–26 were taken into consideration by the European Medicines Agency and resulted in the extension of indication of the quadrivalent vaccine in males 9–26 years of age for the prevention of genital warts.

B) Condoms [74,75] – Grading = 1+, and 2+, respectively

The use of a condom reduces, albeit not completely, the risk of transmission of HPV infection between heterosexual partners. Moreover, correct and consistent use of a condom reduces the risk of transmission by approximately 50%; and appears to promote clearance of the infection as seen on a randomized trial conducted in women diagnosed with CIN lesions and their partners, in the group that used condom, correct usage seemed to promote regression of CIN lesions and clearance of HPV infection on both [74]. Condom usage also reduces the risk of HPV infection in males from female infected partners [75].

#### Question conclusion

Vaccination on males, according to recent data, seems to be effective with the quadrivalent vaccine as it is in women. Vaccination is the most effective measure to reduce HPV related diseases impact in males with efficacy rates around 90%;. Condom usage seems to be also effective in reducing at least in 50%; the risk of transmission when used correctly.

#### Conclusive considerations of the consensus panel

➣ An awareness campaign should be implemented about available vaccines, the importance of vaccination and its high efficacy in males. Also about other prevention measures such as condom usage, through all institutional channels.

➣ The quadrivalent HPV vaccine has proven to be effective in protecting males against genital warts and HPV related precancerous anal lesions. Based on vaccine efficacy results, male vaccination should be recommended as in females as they can benefit too from vaccination.

### Question no. 4: Could primary prevention of HPV-related diseases in men reduce HPV-related diseases in their partners?

A) Vaccination [76] – Grading = 2–

Little data is still available about vaccination effect on a population level. Only on the years to come data will become more available and valid. Only one ecological study, obtained from vaccination programs carried out in women in Australia, were vaccination coverage is high on women, although still early have shown that vaccination induces also some herd immunity in men i.e. there is a partial reduction in the occurrence of anogenital warts in heterosexual males compared to vaccinated women. In Australia, the quadrivalent vaccine has been administered to schoolgirls aged between 12 and 18 years and to women younger than 26 years since 2007. The coverage rate in the area where the study was conducted ranged from 65%; to 75%;. One year after the implementation of the mass vaccination program, comparison of the rates of diagnosis of anogenital warts before and after the introduction of vaccination showed a 48%; reduction in the diagnosis of anogenital warts in women younger than 28 years, but no corresponding reduction was obtained in women older than 28 years and in the males who have sex with males population [76].

Over the same period, a 17%; reduction in the diagnosis of genital warts among heterosexual males was recorded. This reduction was not observed in homosexual males, a finding that suggests some herd immunity effect. In other words, use of the vaccine confers indirect protection on the unvaccinated population as a result of the reduction in the total number of infected subjects, which in turn decreases the number of potentially infectious contacts.

B) Reduction in HPV-related events [77,78] – Grading = Publications could not be assessed, because they are abstracts or other types of communication

In view that natural history in males, although explained, still lacks further research as today we know that although similar, it does not behave as in females and because of the paucity of established epidemiological data, it is impossible (at the moment) to provide details on the impact of a primary prevention strategy on the reduction of HPV-related disease in partners

The complexity of the scenario produced by the implementation of primary prevention in males is due to a number of general considerations: 1) there is limited information on the natural history of HPV infection in males, that behaves differently than females (incidence, prevalence, seroprevalence and burden of disease is different), information is still scarce and more studies are still needed; 2) the inconsistencies of epidemiological data and rates of concordance of genotypes among couples (which are in turn modulated by differences in sexual behavior, age, relationship time and by the diagnostic method adopted among other factors); 3) The lack of a reliable and validated monitoring and diagnosis system (in the long and short term) for males.

Given that HPV infection is a sexually transmitted disease, interventions for primary prevention in one partner will evidently have a positive effect in terms of reducing HPV-related disease in the other partner. Mathematical models indicate that the cost–effectiveness ratio for the vaccination of males becomes favorable in the presence of a low coverage rate for females, because it can contribute towards the containment of infection in the community [77,78], some even state that it becomes more cost effective to vaccinate males rather than hard to reach women [79]. As a result, herd immunity from vaccinating only women is likely to be insufficient to eradicate HPV infection, in fact, on the other hand, a single sex HPV vaccination campaign may also increase the psychological burden on women [80], and this sex inequality could amount to an additional healthcare burden. As a disease that affects both men and women, social fairness needs to be taken into consideration as both individually can benefit from the vaccine. There’s also the matter of males who have sex with males, to whom no benefit is gained from female vaccination only as shown in early ecological studies [76]. In addition, lessons learned from history, seem to show that single gender vaccination campaigns, such as the one against rubella in 1996, that although both diseases differ significantly, there are several potential pitfalls in single sex vaccine programs [81]. From this perspective, it remains to be seen whether the current objective of the vaccination program in Italy will be achieved, namely 95%; coverage with three doses of vaccine within 5 years from the start of the program. The available data for the 1997 born cohort (updated to 31 December 2009) indicate that the coverage rate at 3 vaccine doses is only 53.1%; [82].

#### Question conclusions

Although herd immunity exist, primary vaccination exclusively of males or females doesn’t seem to be effective when coverage rates are less than high in reducing HPV related disease on partners in a significant way. In Italy coverage rates are not high, only 53%; of 12 years old females have been vaccinated with 3 doses. Social equity right to get the vaccine in males and higher risk groups, as well as social burden (including psychological burden) should be taken into consideration as they seem relevant.

#### Conclusive considerations of the consensus panel

➣ When vaccination campaigns targets only one gender, vaccine coverage should be very high in order to expect significant effects in the other gender morbidity.

➣ Were possible, vaccine programs to both genders should be implemented as they seem to be more effective.

➣ More initiatives should be directed towards the population raising awareness of primary prevention, in order to achieve high vaccination coverage in the already implemented vaccination campaign in females to guaranty at least some herd immunity in males.

➣ Specific efforts should be made to vaccinate higher risk groups such as immunosuppressed subjects.

## Conclusions

### Consensus conference statements and final remarks

On the evidence presented by the 4 main questions made to the SC by the SP, the Jury composed by the multidisciplinary consensus panel agrees that HPV (human papillomavirus) impact is important in males. Based on the specific scientific data analyzed and the conclusive considerations the multidisciplinary consensus panel made, they conclude the following recommendations:

#### Recommendations made by a majority

➣ For prevention purposes and social fairness, 12-year-old males should be vaccinated. The jury did not deal with aspects related to healthcare policy and economic sustainability, which are to be discussed elsewhere by others.

#### Unanimous recommendations

➣ The Regions of Italy should introduce an HPV vaccination program with facilitated access (reduced price) for males, as they have done already for females.

➣ More initiatives should be directed towards the population raising awareness of primary prevention and disease. As a result, vaccination coverage rates may improve in the ongoing vaccination campaign in females to indirectly reduce some of male’s morbidity.

➣ Specific efforts should be made to vaccinate groups at higher risk of infection and HPV disease development such as immunosuppressed subjects, especially HIV + .

➣ Awareness campaigns of new scientific evidence should be implemented not only for anogenital warts but for HPV-related diseases in general, through all institutional channels.

➣ Further research on the issue of HPV and fertility worldwide is required in order to fully understand if it is really caused by HPV and in what measures infertility rates can be reduced by HPV prevention measures such as vaccination.

➣ Prevalence and incidence studies in males should be further conducted. In Italy, notification of HPV related diseases such as genital warts are not mandatory, more epidemiological data is needed in order to evaluate disease burden.

➣ Scientific studies should be conducted about the standardized collection of biological samples and testing methods used for HPV detection in males in order to implement in the future standardized guidelines on sampling and diagnostic tests in males.

## Competing interest

The study was supported by an unrestricted funding from Sanofi Pasteur MSD, Italy.

## Pre-publication history

The pre-publication history for this paper can be accessed here:

http://www.biomedcentral.com/1471-2458/13/117/prepub
